# The effects of family directed power mobility on self-care, mobility, and social function in very young children with severe multiple developmental impairments

**DOI:** 10.3389/fresc.2025.1551536

**Published:** 2025-02-25

**Authors:** Juan Aceros, Guilherme M. Cesar, Ayshka Rodriguez, Mary Lundy

**Affiliations:** ^1^ School of Engineering, University of North Florida, Jacksonville, FL, United States; ^2^ Department of Physical Therapy, University of North Florida, Jacksonville, FL, United States

**Keywords:** power mobility, children, developmental disabilities, functional skills, caregiver assistance

## Abstract

**Introduction:**

Children with impaired mobility often experience negative impact on overall development leading to depression, social isolation, and perceived lower quality of life.

**Objective:**

Our study explored the effects of Power Mobility Devices (PMD), in the form of modified ride-on toy cars with two distinct activation/steering technologies, on functional independent and social function in young children with severe multiple developmental impairments.

**Methodology:**

Twelve children (age range 12–54 months) with neuromuscular, musculoskeletal, and genetic diagnoses, and metabolic progressive diseases participated. Significant cognitive, visual, or communication impairment was not exclusionary. Two types of activation and steering modifications (proportional control joystick and line follower technologies) were provided. Paired samples *t*-test contrasted pre-post functional capabilities after three months of PMD use in both Pediatric Evaluation of Disability Inventory scales of Functional Skills and Caregiver Assistance, each with the subscales self-care, mobility, and social/cognitive.

**Findings:**

Improvements were observed in all three subscales for Functional Skills (significant 9.8% increase in self-care, 21.4% in mobility, and 17.5% social/cognitive) and Caregiver Assistance (significant 35.4% increase in self-care).

**Conclusion:**

These quantitative results support findings of previous studies analyzing qualitative data, suggesting that early power mobility interventions provide positive improvements in the quality of life of children with severe developmental disabilities.

## Implications for rehabilitation

• Independent exploration benefits children's cognitive, sensorimotor, social, and emotional development. These benefits are also expected for children with developmental disabilities.
• Power mobility devices (PMD) can help mitigate the negative effects of limited self-directed exploration in children with mobility impairments. However, despite evidence of positive effects, PMD use in young children with disabilities is underutilized.
• Gaining parental approval and securing funding for power mobility are among the most significant obstacles faced by assistive technology professionals. Understanding cause-and-effect relationships is often seen as a prerequisite.
• This study suggests this is unnecessary and that power mobility can aid in learning this concept. Technology can bridge the gap for children with behavioral, motor planning, or processing issues, helping them become mobile despite these challenges.

## 1 Introduction

Independent, self-directed exploration of the environment has been shown to positively impact children's cognitive, sensorimotor, social, and emotional development (1). Although this benefit should also be expected for children with developmental disabilities, participation in independent exploration and play requires motor and sensory functions that are often limited in this population (2–4). Research has shown that inadequate early self-initiated independent mobility and environmental exploration restricts learning and social participation, which can result in a cycle of “learned helplessness”, decreased curiosity, social isolation, depression and a perceived lower quality of life (5, 6).

An approach than can mitigate the negative outcomes associated with the lack of self-directed exploration in children with mobility impairments is to facilitate the use of power mobility devices (PMD) (7). These devices are mechanically-propelled mobility aids, powered by electric motors that are often controlled by a joystick, switches, or other adaptive control systems tailored to the child's specific needs, and are designed to assist those with walking impairments in achieving independent movement (8). Examples of PMW are power wheelchairs or modified Ride-on toys. However, a survey with 424 multidisciplinary early intervention providers indicated that PMD use in young children with disabilities is underutilized and rarely recommended (9) based on the assumption that it will impede acquisition of motor skills, particularly independent ambulation. Despite the growing body of evidence suggesting that PMD use with very young children has positive effects without causing deterioration of existing motor skills or interfering with emerging new ones (10–12), the standard of practice for not prescribing power mobility to children with disabilities continues.

The lack of recommendation for PMD at an early age has left a void in technology options for young children with disabilities. Recent attempts to accommodate such void has been explored, such as the Wizzybug and the Cub (13) and the Permobil Mini Explorer [Permobil AB, Timra, Sweden] (14), which, to our knowledge, is the only commercially available PMD for children 12–36 months. While these technologies are still emerging and the quantification of their impact on children's motor control and development is still scarce, devices traditionally prescribed for children with mobility impairment are power wheelchairs. However, in addition to high costs associated with power wheelchairs, the common clinical practice is to prescribe such devices only for children older than 3 years (15), restricting the positive effects of early independent, self-directed exploration of children's development.

An alternative PMD option provided through the community outreach program Go-Baby-Go has gained recognition as a means for cost-effective introduction and extended experience with power mobility for very young children with impaired mobility. Even though increase in socialization and functional mobility were reported in young children with various motor abilities (16, 17) after use of this PMD in the form of battery powered modified ride-on toy car (18), these findings originated from series of single case studies only. Furthermore, a key modification to the PMD was based on a large single push button activation switch placed on the steering wheel. This modification proved difficult for children with hand dexterity impairments to achieve directional control and slow acceleration speeds (18). Given that the ability to control PMD's directional path is essential for goal-directed, self-initiated autonomous locomotion, this steering modification can be improved to address upper extremity capabilities to allow for optimal exploration and interaction with the environment (19).

When considering young children with severe impairments, literature suggests two types of activation and steering modifications that could facilitate PMD use, the *proportional control joystick* and *line follower technologies* (20). While steering with the joystick can be learned by infants as young as 9 months old (21) (7) and also by those with severe cognitive impairments, the line follower technology also enhances PMD usability to those with visual impairments since the ride-on car safely follows a marked line on the ground using a series of optical sensors. Therefore, the objective of our study was to explore the effects of PMD modified with these two activation/steering technologies on functional skill performance and level of independence in children under five-years of age with severe multiple developmental impairments. Given the focus on the young age, we also focused on exploring the impact of the modified PMD on caregiver assistance on activities of daily living.

## 2 Methods

This section outlines the study's design, which involves a 3-month period of intervention with modified ride-on toy cars and pre-post evaluation of functional outcomes. The following sub-sections include participant recruitment, screening details, standardized measures, study visits, modifications to PMDs, safety protocols, training procedures, and statistical analysis. The Participants section explains the selection criteria, recruitment process, and demographic details of the children who took part in the study. The Screening section details the assessment framework and criteria used to evaluate the children's abilities. The Standardized Measure section describes the Pediatric Evaluation of Disability Inventory (PEDI) used to assess functional outcomes. The Study Visits and Customization Process section explains the steps taken during the study visits, including the design and customization of the ride-on toy cars. Modifications discusses the specific adaptations made to the cars to meet each child's needs. Safety outlines the safety protocols and training provided to families. Training describes the initial familiarization and usage (i.e., intervention period) of the PMDs by the children. Finally, the Statistical Analysis section presents the methods used to analyze the data collected from the study.

### 2.1 Participants

Participants included children diagnosed with multiple developmental disabilities with significant mobility impairments. Pediatric physical therapists working in rehabilitation outpatient facilities, early intervention programs, and public-school systems in the northeast Florida identified these children as needing access to power mobility and introduced parents/legal guardians to our program. Parents contacted the University and were invited to schedule a get acquainted visit with their child and the project personnel. A welcome packet was physically handed to the parent/guardian of all children during their visit. The welcome packets contained a brief explanation of why play is important for all children, a summary of the program, background information for faculty, contact information for the project, a health history form for the parent/guardian to fill out to help decide on an appropriate toy and needed adaptations to the toys, and a recruitment flyer. Every effort was made to reassure parents and the child that regardless of their willingness to participate in the study, their child would receive an appropriately adapted toy through the program. To minimize potential feelings of undue influence, parents/guardians were reassured that the primary goal of the project is to make sure that adaptive toys are accessible to children with disabilities in our community, and not the research study.

Eighteen children aged six months to five years were referred for possible enrollment in this study. Children were not excluded from enrolling for any reason. Consent was obtained from each participant's parent/legal guardian as approved by the Institutional Review Board. Six children who had been referred to the program did not enroll in the study due to parental job relocation, moving out of state for personal reasons, or by request from a foster agency due to current litigation. However, all children received a modified ride-on toy. The enrolled study sample consisted of 12 children with neuromuscular diagnosis, musculoskeletal, or genetic and metabolic progressive diseases. Significant cognitive, visual, or communication impairment was not exclusionary. The final cohort included both males and females ranging in age from 12 to 54 months (mean = 32 months). Several children had the diagnosis of Cerebral Palsy, with varying levels of severity (III–V), while one child had Arthrogryposis and another a progressive neurological/muscular disease. The children's overall abilities varied, with some having age-appropriate skills and others experiencing delays. Communication methods also differed, with some children being verbal, others using gestures, and one utilizing an augmentative system. Coordination issues, such as dysmetria, were common among the children. Vision capability varied, in which some children displayed normal or corrected vision, while others had cortical visual impairment (CVI). Despite these challenges, many of our participants exhibited emerging skills and abilities. Detailed information is presented in Table 1.

**Table 1:** Participant demographics and screening results.

Participant	Age (months)	Sex	Diagnosis	GMFCS	Cognitive level	Communicative Ability	Follows directions	Understands cause & effect	UE reaching	Vision
1	12	Female	Congenital Brain Malformation	NA	Delayed	Non-verbal uses Gestures	Emerging	Emerging	Poor/Dysmetria	Normal (corrected), Glasses
2	13	Male	Non-Accidental TBI	V	Delayed	Non-Verbal does not gesture	No	No	Does not reach for objects	Significant visually impaired
3	18	Female	Progressive Neurological/Muscular Disease	NA	Age appropriate	Non-verbal uses Gestures	Yes	Yes	Good	Normal
4	24	Male	Arthrogyrposis	NA	Age appropriate	Verbal	Yes	Yes	Poor/Dysmetria	Normal
5	30	Male	Cerebral Palsy	IV	Delayed	Non-verbal uses Gestures	Yes	Yes	Poor/Dysmetria	Normal (corrected), Glasses
6	36	Male	Cerebral Palsy	IV	Age appropriate	Non-verbal uses Gestures	Emerging	Yes	Poor/Dysmetria	Normal (corrected), Glasses
7	36	Female	Cerebral Palsy	III	Delayed	Non-verbal uses Gestures	Emerging	Yes	Good	Normal (corrected), Glasses
8	36	Male	Cerebral Palsy	V	Delayed	Non-verbal uses Gestures	Emerging	Emerging	Poor/Dysmetria	CVI
9	36	Female	Cerebral Palsy	V	Delayed	Non-verbal uses Gestures	Emerging	Emerging	Poor/Dysmetria	CVI
10	36	Male	Cerebral Palsy	IV	Delayed	Non-verbal uses Gestures	Emerging	Emerging	Poor/Dysmetria	CVI
11	48	Male	Cerebral Palsy	V	Delayed	Non-verbal uses Gestures	No	Emerging	Poor/Dysmetria	CVI
12	54	Male	Progressive Neurological/Muscular Disease	NA	Age appropriate	Augmentative system	Yes	Yes	Poor/Dysmetria	Normal

### 2.2 Screening

The International Classification of Functioning, Disability and Health for Children and Youth (ICF-CY) (22) served as a framework for assessment in this study. Each child was screened by the referring physical therapist in the areas of cognition, communication, sensory abilities, strength, and upper extremity coordination, range of motion, muscle tone, sitting balance and independent mobility. Also, the Gross Motor Function Classification System (GMFCS) level was assigned to children enrolled in the study with the diagnosis of cerebral palsy (23). This information was provided to the researchers through the intake sheet and verified by parental report and evaluation by the physical therapist researchers on the participants first visit. In addition to the screening, each child was measured using basic adaptive seating fundamental principles including proximal stability, skeletal alignment, stable base of support, and sitting position to facilitate function (24). The participant demographics and screening results are shown in Table 1.

### 2.3 Standardized measure

The standardized functional measure selected was the Pediatric Evaluation of Disability Inventory (PEDI). The PEDI is a functional outcome measure with the high degree of responsiveness to detect changes in children with severe multiple developmental impairments. PEDI has established concurrent and construct validity for children with disabilities and it is valid and reliable for children aged six months to 7.5 years with varied diagnoses including severe physical and/or cognitive impairments (25). This tool measures functional skill performance and level of independence in three domains through caregiver report, Self-Care, Mobility, and Social Function. In this study, all three domains were evaluated in two sets of scales: Functional Skills and Caregiver Assistance. The Functional Skills scale contains 197 items that relate to activities of daily living and are scored from zero (*unable to perform*) to one (*capable of performing*). The Caregiver Assistance scale contains 20 items and assesses the amount of help a child requires to complete these activities, scored in a ratio scale from zero (*total assist*) to five (*independent*). The total raw scores for each subscale were converted to scale scores (0–100) (26).

### 2.4 Study visits

Each child participated in three visits to the university for this study. During the first visit all required forms and consents were obtained for enrollment, services and dissemination of study results. A pre-intervention PEDI was administered by the physical therapist researchers on the participants’ first visit and the technology design-fabrication team (consisting of a physical therapist, a mechanical engineer, and an electrical engineer) completed specific measurements similar to those taken when prescribing a wheelchair seating system. A design for a battery-powered modified ride-on toy car was then created based on the general screen completed by the referring therapist and specific measurements obtained by the technology design-fabrication team.

The second visit consisted of PMD delivery, safety and training. More details regarding his visit are available in the safety and trainings sections of this manuscript. The third and final visit consisted of post intervention and questionnaire response by caregivers. It was reported at this time that all children were using their PMD. The frequency of use was recorded via a qualitative data response questionnaire with caregivers reporting ranges from one-two times/week to everyday, most averaging two-three times/week. In addition, one child reported its use at school, one as part of inside activities and all others outside. This reported high adherence to PMD use is attributed to lower barriers (space, weather, terrain) found in the North Florida area where the study took place. It is also attributed to the selection and design on the ride-on toy cars using a 12-volt battery which allowed it to be operated over rougher outdoor terrains. The cars were used indoors and outdoors for play with other children, for participation in family walks, and engagement with other children at school and on playgrounds. Lastly, caregivers reported a perceived increase in spatial awareness, cause and effect, attention to task and overall happiness.

### 2.5 Modifications

A commercially available ride on toy car with a 12-volt battery was selected for each participant. The battery voltage was selected to allow operation over multiple terrains, e.g., for optimal exploration and interaction with the environment, but also provides the power for smooth acceleration of both proportional joystick control and line follower technologies. The smooth acceleration of the motor is important for children with poor posture control, decreased upper extremity control, and a retained startle reflex. The design modifications for each car were divided into (1) seating and mechanical support for the torso, upper and lower extremities including the pelvis, and the head and neck, and (2) steering and activation mechanisms that included the electrical switching and drive system. Some of the seating and mechanical support modifications included raised seatbacks, head supports, chest straps, pelvic straps, a pelvic lap belt, and lateral trunk supports pads. Common of-the-shelf components were employed including 1-inch Schedule 40 PVC pipe, foam pads, pool noodles, neoprene rubber, EVA foam kickboards, waterproof fabric, and 5-point straps. There are several published articles and how-to guidelines that illustrate the process to complete such mechanical modifications (27–30).

The steering and activation technologies selected were either the “Line follower Technology” for two children with developmental disabilities and significant Cortical Visual Impairment (CVI) or the “Proportional Control Joystick Technology” for 10 children without CVI. A detailed description of these technologies, and their development has been previously reported elsewhere (31, 32). The placement of the activation interface was determined by upper extremity range of motion, coordination, and postural reflexes. The different seating and mechanical support modifications, activation interface location, and the type of activation and steering technology selected for each participant is presented in Table 2.

**Table 2:** Power mobility device modifications.

Participant number	Activation and steering mechanism	Activation, location	Seating and mechanical support	Other
1	Proportional control Joystick	Center	High Seat Back, Head Support, Chest strap, Pelvic Belt	Potentiometer for speed control
2	Push Button	Center	High Seat Back, Head Support, Chest strap, Pelvic Belt, Lateral supports, Abductor pads, 90 degrees hips	None
3	Proportional control Joystick	Center	High Seat Back, Chest strap, Pelvic Belt, Adductor pads, 90 degrees hips	Zero turn radius with hidden casters. Rubber wheels were added for wooden floors at home. Basket on the back for baby doll.
4	Proportional control Joystick	Center	High Seat Back, Chest strap, Pelvic Belt, Adductor pads, 90 degrees hips, Plexiglas tray for support of UE with joystick mounted through the tray	NA
5	Proportional control Joystick	Center	High Seat Back, Chest strap, Pelvic Belt, 90 degrees hips	Music, flashing LED's to the windshield of the car.
6	Proportional control Joystick	Right Side	High Seat Back, Chest strap, Pelvic Belt	Music with activation
7	Proportional control Joystick	Right Side	High Seat Back, Chest strap, Pelvic Belt, 90 degrees hips	The small joystick ball was replaced with Olaf's head to make a larger item for easy grasp
8	Proportional control Joystick	Left Side	High Seat Back, Head Support, Chest strap, Pelvic Belt, Lateral supports, Abductor pads, Tilt in space seat, 90 degrees hips	None
9	Push button	Center	High Seat Back, Head Support, Chest strap, Pelvic Belt	None
10	Proportional control Joystick	Center	High Seat Back, Head Support, Chest strap, Pelvic Belt, Lateral supports, Abductor pads, 90 degrees hips	None
11	Proportional control Joystick	Left Side	High Seat Back, Head Support, Chest strap, Pelvic Belt, Abductor pads, 90 degrees hips	Music and Lights with activation
12	Proportional control Joystick	Right Side	High Seat Back, Head Support, Chest strap, Pelvic Belt, 90 degrees hips	None

### 2.6 Safety

A second visit was scheduled 3 months later for delivery of their modified ride-on toy car. This visit lasted between 1.5 and 2 h and included the safety overview and training operation of the modified ride-on. Safety of the device was paramount and a *Safety Assurance Plan* consisting of an inspection and operation procedures was created and approved by the Institutional Review Board. This plan was a two-part process. First, an expert engineer conducted a safety inspection, which followed a standardized checklist that included visual inspections of the mechanical and electrical modifications to the ride-on and an operational check with a 40-pound load at continuous speed for 10 min. The expert engineer then signed a safety check statement.

The second step of the safety plan included family education. The physical therapist and an engineer met with the families to personally direct them in the operation, charging, safety and care of the modified PMD. The family also received a written manual with this information and contact information in case there was a problem or question. The physical therapist demonstrated the use and adjustment of harnesses, belts, and correct positioning of child in the car. As part of this session the families were required to demonstrate what was learned, including the procedures for charging the battery and operation of a remote stop safety switch that was provided for each car.

### 2.7 Training

**Figure 1:**
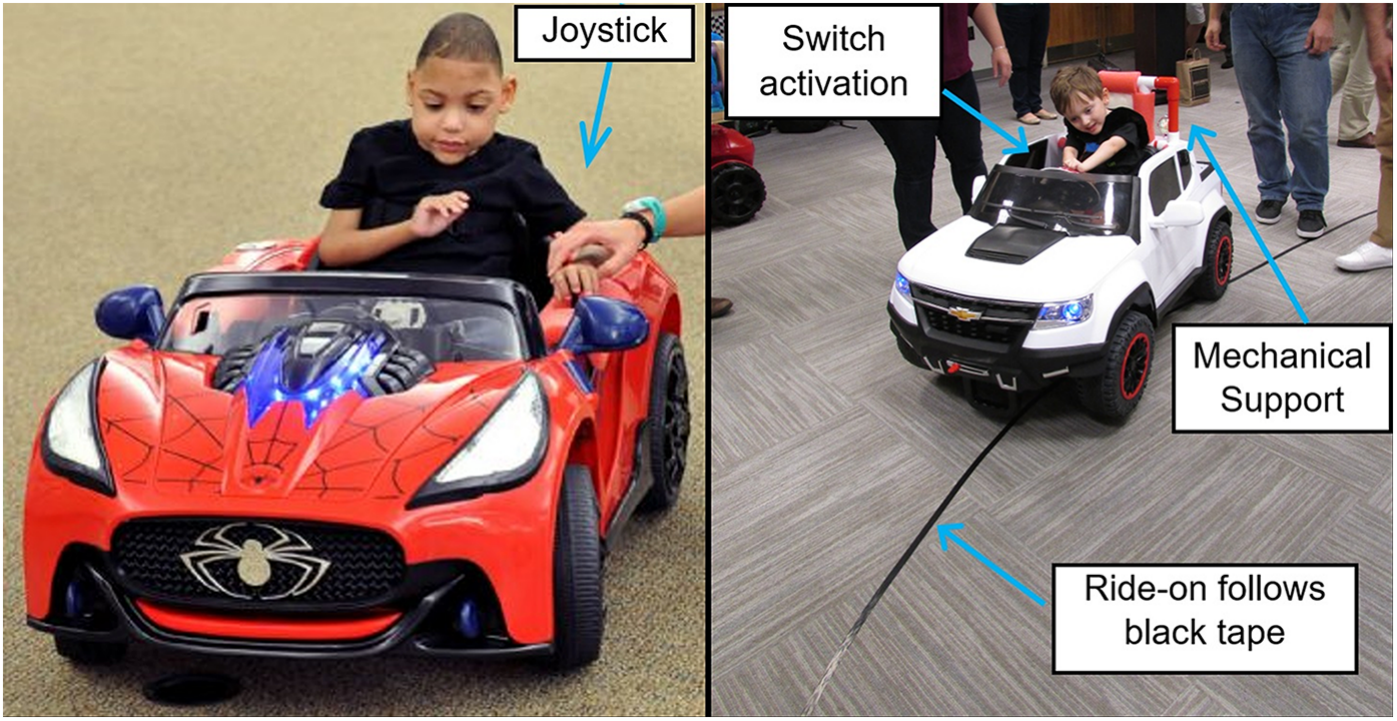
Left: ride-on with proportional control joystick for a four-year-old child with spastic quadriplegia cerebral palsy. Right: Child with Cortical Visual Impairment operates an adapted ride-on with line follower technology.

Once the family received the safety instructions, the child was seated in the car and allowed to become familiar with it. Training consisted of hand-over-hand guidance to briefly demonstrate to the child the cause-and-effect relationship between the activation interface and car movement. Simple one-word verbal labels were used in conjunction with the haptic guidance, e.g., Go, Stop, and Push. After the demonstration, the child was then given the opportunity for random, free exploration of the motor activation and the consequent movement of the car without further adult instruction. Figure 1 presents this training procedure for both types of PMD technologies.

Participants were observed throughout the duration of their initial experience in their powered modified ride-on car, which lasted approximately an hour and a half. All 12 children were able to activate the modified ride-on car and experience the subsequent movement. At the end of the initial experience their activation became intentional and self-initiated. The participants would activate the car, stop, and then repeat the activation. Parents/legal guardians were instructed to integrate the PMD as a play activity into the regular family routine in natural environments. The utilization (frequency/duration of use) of the PMD was not prescribed as a therapeutic intervention. Much like other play activities, the interaction with the PMD was to be self-initiated and self-directed by the child.

It is noted that in this study children approached power mobility as part of “play”, meaning voluntary, enjoyable, process-oriented (not motivated by specific goals), and intrinsically driven by the child. The term “family-directed” in this context emphasizes the active involvement of the family to facilitate activities in a family-oriented environment rather than a clinical environment. This approach aims to mimic normative developmental activities, ensuring that the interventions are aligned with typical childhood experiences and promoting natural development.

### 2.8 Statistical analysis

All pre and post Self-Care, Mobility and Social/Cognitive data sets satisfied assumptions of normality via the Anderson–Darling Normality test (Minitab LLC). Paired samples *t*-test was conducted on each subscale of PEDI to assess changes post PMD experience. Statistical significance level for all comparisons was set *a priori* at alpha value of 0.05. Furthermore, the absolute effect size was tested to help understand the magnitude of differences for each data set and classified as small (≤0.2), medium (0.5) and large (≥0.8) effect (33).

## 3 Results

Twelve children aged 12–54 months with severe multiple developmental impairments were enrolled in this PMD-study. Ten were provided with PMDs with joystick-operation and two with push-button operated line follower technology. The post intervention PEDI assessment was completed during the final third visit, six months after the pre-intervention assessment and three months after delivery of the PMD.

Results from pre and post intervention PEDI are presented for each participant in Tables 3, 4 for Converted Functional Skills and Converted Caregiver Assistant Scale Scores, respectively. Note that higher scores indicate higher function.

**Table 3:** Converted functional scale scores.

Participant	Assessment	Self-Care	Mobility	Social/cognitive
1	Pre	29.4	11.4	30
	Post	36	11.4	32.9
2	Pre	21.4	18.2	10.5
	Post	24.1	20.9	14.7
3	Pre	42	32	41.8
	Post	46	38.2	49.7
4	Pre	37	33.4	45.6
	Post	39.6	38.2	56
5	Pre	21.4	44.3	37
	Post	31.9	44.3	39.6
6	Pre	37	25.4	46.2
	Post	40.4	34.7	50.3
7	Pre	42	34.7	21.6
	Post	42	58.2	36.1
8	Pre	38.7	18.2	34
	Post	42	18.2	52
9	Pre	17.4	20.9	37.9
	Post	17.4	20.9	40.4
10	Pre	17.4	15.2	35.1
	Post	21.4	15.2	38.8
11	Pre	35.1	20.9	40.4
	Post	37.1	29	43.1
12	Pre	51	23.3	57.9
	Post	51	32	62.3

**Table 4:** Converted caregiver assistant scale scores.

Participant	Assessment	Self-Care	Mobility	Social/cognitive
1	Pre	20.1	N/A	N/A
	Post	21.4	N/A	N/A
2	Pre	39.3	36.9	35.9
	Post	39.3	36.9	35.9
3	Pre	20.1	20.1	26.6
	Post	41.1	25.4	55.3
4	Pre	25.4	31.9	89.9
	Post	37.2	54.8	100
5	Pre	11.6	25.4	42.9
	Post	39.3	47.2	45.8
6	Pre	39.3	40.9	57.3
	Post	39.3	44.3	61.3
7	Pre	39.3	58.8	11.3
	Post	48.6	54.8	50.9
8	Pre	11.6	N/A	39.6
	Post	25.4	N/A	45.8
9	Pre	N/A	N/A	N/A
	Post	N/A	N/A	20.4
10	Pre	39.3	39	35.9
	Post	39.3	34.5	35.9
11	Pre	20.1	29	20.4
	Post	20.1	29	39.6
12	Pre	32.3	42.7	78.6
	Post	53.4	11.7	67.6

The changes observed as a cohort are summarized in Table 5 below. Significant improvements were observed for all subscales in Functional Skills. Only the subscale Self-Care significantly increase for Caregiver Assistant even though all subscales exhibited increased scores.

**Table 5:** Summarized scores of pediatric evaluation of disability inventory.

PEDI Subscales	Functional scale				Caregiver assistant scale			
	Pre average (SD)	Post average (SD)	*p*	Effect size	Pre average (SD)	Post average (SD)	*p*	Effect size
Self-care	32.5 (11)	35.7 (10)	0.003	0.3	27.1 (11)	36.7 (10)	0.011	0.9
Mobility	24.8 (9)	30.1 (13)	0.003	0.4	36.1 (11)	37.6 (14)	0.778	0.1
Social/cognitive	36.5 (12)	42.9 (12)	0.001	0.5	43.8 (25)	53.8 (19)	0.066	0.4

## 4 Discussion

It is well-established that children's overall development is impacted by the ability to explore the environment through self-initiated, self-directed locomotion. Previous work with pediatric wheelchair prescribers demonstrated that cognitive impairment and sensory issues, like cortical visual impairment, were common reasons for not recommending PMDs for children (34). An additional barrier was funding denials due to lack of proof of a successful power mobility trial, which requires demonstrating cognitive skills like cause and effect, spatial awareness, and safety judgment (35, 36). While children with severe mobility impairments may lack these skills initially, exposure to and practice with PMDs can help children become proficient (8, 9, 11), highlighting the potential need of providing PMD access for this population to promote overall development, independence, and participation in life activities. Given the limited ability for independent mobility in children with developmental disabilities, we focused on understanding whether PMD use could promote a positive impact on functional performance and independence in young children with multiple developmental impairments. Our findings suggested that allowing for self-directed mobility via adapted PMDs improves quality of life in children and caregivers. All children in the study successfully learned to operate their PMDs (10 children for joystick and 2 for liner follower). One interesting observation with the joystick operation was that all the children first pulled the joystick towards them, which initiated backward movement before learning to push the joystick to move the car forward.

Interestingly, the two participants who engaged with the modified PMD via push button/line follower did not exhibit the same meaningful functional differences as observed with other participants who utilized proportional control joystick. Although the cohort is heterogeneous in itself, the characteristics of both children (participants 2 and 9) do not deviate from the distribution of characteristics among other participants. Considering the use of the activation and steering technology alone, it appears that the use of the joystick may require better interaction with the PMD and cause-effect relationship explorations that do not emerge with the push button/line follower. However, a relationship cannot be established with our study design and future work should further explore this activation strategy. The following provides a discussion on Self-care, Mobility, and Social Function.

### 4.1 PEDI-related outcomes

The Self-Care function subscale assesses tasks related to activities of daily living requiring upper extremity coordination and strength, including eating, grooming, bathing, dressing, and toileting. Similarly, upper extremity control, range of motion, and strength are necessary to engage with and activate the interface devices that control the adapted PMDs. In our cohort, 75% of the participants demonstrated an increase in self-care based on the Functional Skills scale score, a finding in alignment with prior work with power wheelchairs for young children with disabilities (7, 8). Important to highlight that this improvement also impacted the children's family as the 35.4% increase in Caregiver Assistant score reflects lesser caregiver burden and improved independence.

The Mobility function subscale assesses two basic types of mobility, including basic transfer skills (e.g., getting in and out of a chair) and body transport skills (e.g., floor mobility, locomotion). While no statistically significant change was observed in the Caregiver Assistant scale, the Functional Skills scale score showed a significant positive change for our participants, with one child with cerebral palsy exhibiting a 68% increase in this subscale. This result is of importance given the transitional skills of this subscale require movement through space and therefore postural control and well-coordinated oculomotor-vestibular reflexes. In addition, self-initiated movement through the environment activates the visual processes by providing stimulation of the vestibular system and can improve vision in children with CVI as well as balance (37). This study included four children with CVI and one child with a significant visual impairment. Two of these children increased mobility function post-intervention, similarly to studies where children with CVI and severe motor impairments learned to operate PMDs and improved mobility skills (38, 39).

The Social Function subscale evaluates skills necessary for community living, such as participation in family activities that encompass understanding of instructions, articulating information, joint problem solving, and peer play. Even though only a moderate support has been reported for the impact on young children's participation and social interactions after PMD use (8), every participant in our study exhibited gains in this subscale, with three children surpassing the minimally clinically important difference of 10 points (40). This positive increase can be attributed to the direct relationship between social function and the ability to play—which was the basis for providing PMD in this study—but also to the nature of the PMD, i.e., Adaptive Ride-On Toy, which increases the child's desire to engage and manipulate objects with intent.

### 4.2 Applicability of overall findings

The results of our quantitative study support findings of the qualitative literature (the only type of analysis performed to date) regarding the impact of power mobility on children with motor disabilities and their families. Although the greater cohort of children (1) exposed to PMD were over four years old, analyzes identified three overarching themes in alignment with our findings, including that power mobility promotes independence, increases opportunities for engagement with the environment, and enhances social relationships (1).

All children from our study learned to operate their ride-on using either a joystick or line follower technology. It is noted that children were able to self-initiate movement using a joystick interface in a short period of time through free exploration without specific training. The basic operational mechanism of proportional control employed by joystick technology is similar to the one employed by power wheelchairs, suggesting that young children with severe multiple disabilities at early developmental levels can learn to operate power wheelchairs. Furthermore, the level of severe disabilities exhibited by our participants presents great clinical implications since caregivers of children with GMFCS level V reported numerous positive changes in children's participation and development that were similar to the changes observed for those at lower GMFCS levels (41).

This information could support a potential shift in standard practice patterns and result in PMD being provided during critical neurodevelopmental periods for optimum impact (42). Our findings expand on previous research (12, 17, 27, 43, 44) and is a strong indicator that early power mobility interventions can enhance the quality of life of children with severe disabilities, including those with visual impairments. Such early access could potentially have long-reaching implications including impacting future independent functional prognosis, medical management, educational cost, academic success and overall quality of life for children with disabilities.

### 4.3 Applicability for clinical care

Among the many obstacles faced by assistive technology professionals, gaining approval from parents and securing funding stand out as two of the most substantial challenges. Funding sources have exhibited reluctance to support power mobility due to safety concerns, such as the child's capability to operate the device while comprehending cause-and-effect relationships. Given that many clinicians and payors consider understanding this relationship to be a prerequisite for power mobility, we recommend re-evaluating this condition. Our project demonstrated that it is unnecessary. In fact, power mobility can provide a faster learning platform for understanding cause and effect, as the sensory immersion of moving through space fosters greater engagement. This does not imply that a child will effortlessly or rapidly master the operation of a PMD; however, even children with significant complexities and very young age as the participants of our study can eventually acquire this skill.

Usual concerns regarding safety often revolve around the child's ability to operate these devices and comply with instructions. However, it's important to note that even typically developing children require supervision when using power mobility (10). In our research, many children and their families initially utilized open spaces to become acquainted with and learn how to use the PMD before transitioning to indoor environments. Furthermore, while the ability to understand and follow commands can expedite the learning process for operating a PMD, it is not an absolute requirement. It is worth emphasizing that even typically developing children may not consistently follow instructions, and as demonstrated in our study, technology can serve as a bridge between a non-mobile child and one who can independently move though space (18). In other words, if a child's safety or capacity to follow basic commands is hindered by behavioral, motor planning, or other processing issues (e.g., Cortical Visual Impairment), technology can be a valuable aid to overcome this challenge (e.g., line follower) (45).

## 5 Limitations

This study did not control for the possible effect of growth on the performance of participants. It is reasonable to assume that children could improve on the PEDI even if not using a ride-on, as these are years of rapid development. Future studies using a waitlist control design would help assess improvement regardless of intervention. Another limitation of this study is the effect that music or flashing lights may have on encouraging use of the ride-on toy car. These types of adaptation are used to engage the children's interest in the ride-on and may have a larger influence on the child's desire to use a power mobility device than mobility itself. Future work on this is recommended.

In addition, the project employed parental/caregiver feedback to track PMD use (dosing). This form of monitoring suffers from biasing and reliability. Caregivers might overestimate or underestimate dosing due to stress, fatigue, or anxiety or they may also feel pressure to report higher usage than actual reality. An unbiased approach to dose monitoring will greatly improve the reliability of these studies.

## 6 Conclusion

Children with disabilities younger than five years old improved functional capabilities after use of power mobility in the form of modified ride-on toy car. Our findings support current recommendation of power mobility devices for very young children with severe multiple developmental impairments.

## Data Availability

The raw data supporting the conclusions of this article will be made available by the authors, without undue reservation.
